# Potential for NPY receptor–related therapies for polycystic ovary syndrome: an updated review

**DOI:** 10.1007/s42000-023-00460-8

**Published:** 2023-07-14

**Authors:** Wei-hong Chen, Yan-chuan Shi, Qiao-yi Huang, Jia-ming Chen, Zhi-yi Wang, Shu Lin, Qi-yang Shi

**Affiliations:** 1 Department of Gynecology and Obstetrics, The Second Affiliated Hospital of Fujian Medical University, No.34 North Zhongshan Road, Quanzhou, 362000 Fujian Province China; 2 Centre of Neurological and Metabolic Research, The Second Affiliated Hospital of Fujian Medical University, No.34 North Zhongshan Road, Quanzhou, 362000 Fujian Province China; 3 Diabetes and Metabolism Division, Garvan Institute of Medical Research, 384 Victoria Street, Darlinghurst, Sydney, NSW 2010 Australia

**Keywords:** Polycystic ovary syndrome, Neuropeptide Y, Gonadotropin-releasing hormone, Dysgenesis, Metabolic disturbance, Leptin

## Abstract

Polycystic ovary syndrome (PCOS) is a complex endocrine disease that can cause female infertility and bring economic burden to families and to society. The clinical and/or biochemical manifestations include hyperandrogenism, persistent anovulation, and polycystic ovarian changes, often accompanied by insulin resistance and obesity. Although its pathogenesis is unclear, PCOS involves the abnormal regulation of the hypothalamic-pituitary-ovarian axis and the abnormal activation of GnRH neurons. Neuropeptide Y (NPY) is widely distributed in the arcuate nucleus of the hypothalamus and functions as the physiological integrator of two neuroendocrine systems, one governing feeding and the other controlling reproduction. In recent years, an increasing number of studies have focused on the improvement of the reproductive and metabolic status of PCOS through the therapeutic application of NPY and its receptors. In this review, we summarize the central and peripheral regulation of NPY and its receptors in the development of PCOS and discuss the potential for NPY receptor–related therapies for PCOS.

## Introduction

Polycystic ovary syndrome (PCOS)—also known as the Stein-Leventhal syndrome which was first reported by Stein and Leventhal in 1935 [1, 2]—is the most common endocrine disease among women of childbearing age. According to the current diagnostic criteria, the global prevalence of PCOS is 4–21% [3, 4]. Patients with PCOS have various clinical sequelae that are serious in nature, including severe mental health problems (e.g., reduced quality of life, poor self-esteem, depression, and anxiety), reproductive complications (infertility and pregnancy issues), and metabolic implications (insulin resistance (IR), metabolic syndrome, and diabetes). Due to the heterogeneity and clinical characteristics of PCOS, its course may vary throughout a person’s lifetime [5]. At present, the most common treatment options for PCOS include lifestyle changes (especially diet and strengthening exercises) and drugs, which help regulate the menstrual cycle, reduce androgen levels, improve IR, and promote ovulation. Treatment for PCOS may also require surgery. These therapeutic options primarily focus on the treatment of symptoms; however, in some cases, they do not produce satisfactory results [6].

Although PCOS has been known for a long time, its pathophysiological mechanism remains unclear. Imbalance of the hypothalamic-pituitary-ovarian (HPO) axis is considered an important pathophysiological mechanism of PCOS [7]. Gonadotropin-releasing hormone (GnRH) neurons project to the median eminence and release pulses of GnRH peptide directly into the pituitary portal vasculature that drive the pulsatile release of the gonadotropin-luteinizing hormone (LH) and follicle-stimulating hormone (FSH) from the pituitary gland [1, 8]. Abnormal GNRH pulse release can lead to an abnormal LH/FSH ratio. Hormone tests in women with PCOS show elevated LH levels. This has also been observed in letrozole-induced PCOS mouse models [9]. Studies have shown that NPY acts directly on GnRH neurons [10, 11] and affects metabolism and reproduction.

Neuropeptide Y (NPY) is a 36–amino acid neuropeptide that is highly conserved among species and is one of the most abundant neuropeptides in the central nervous system of mammals [12–15]. Five mammalian NPY receptors (Y1, Y2, Y4, Y5, and Y6) have been cloned in mammals. The Y1, Y2, Y4, and Y5 receptors are all G-protein coupled. The Y6 receptor is truncated in most mammals (including humans) but is functional in mice [16]. Neuronal NPY participates in the regulation of feeding behavior [17], reproductive behavior [18], energy homeostasis [19], and memory storage [20]. Hypothalamic NPY is an important central regulator of sexual behavior and reproductive functions [21]. In addition, NPY is the strongest appetite-promoting factor in the hypothalamus and controls eating behavior [22]. NPY stimulates appetite, causes overeating, increases body fat, lowers body temperature, and inhibits sympathetic nerve activity [22].

## Pathophysiology of PCOS

**Fig. 1:**
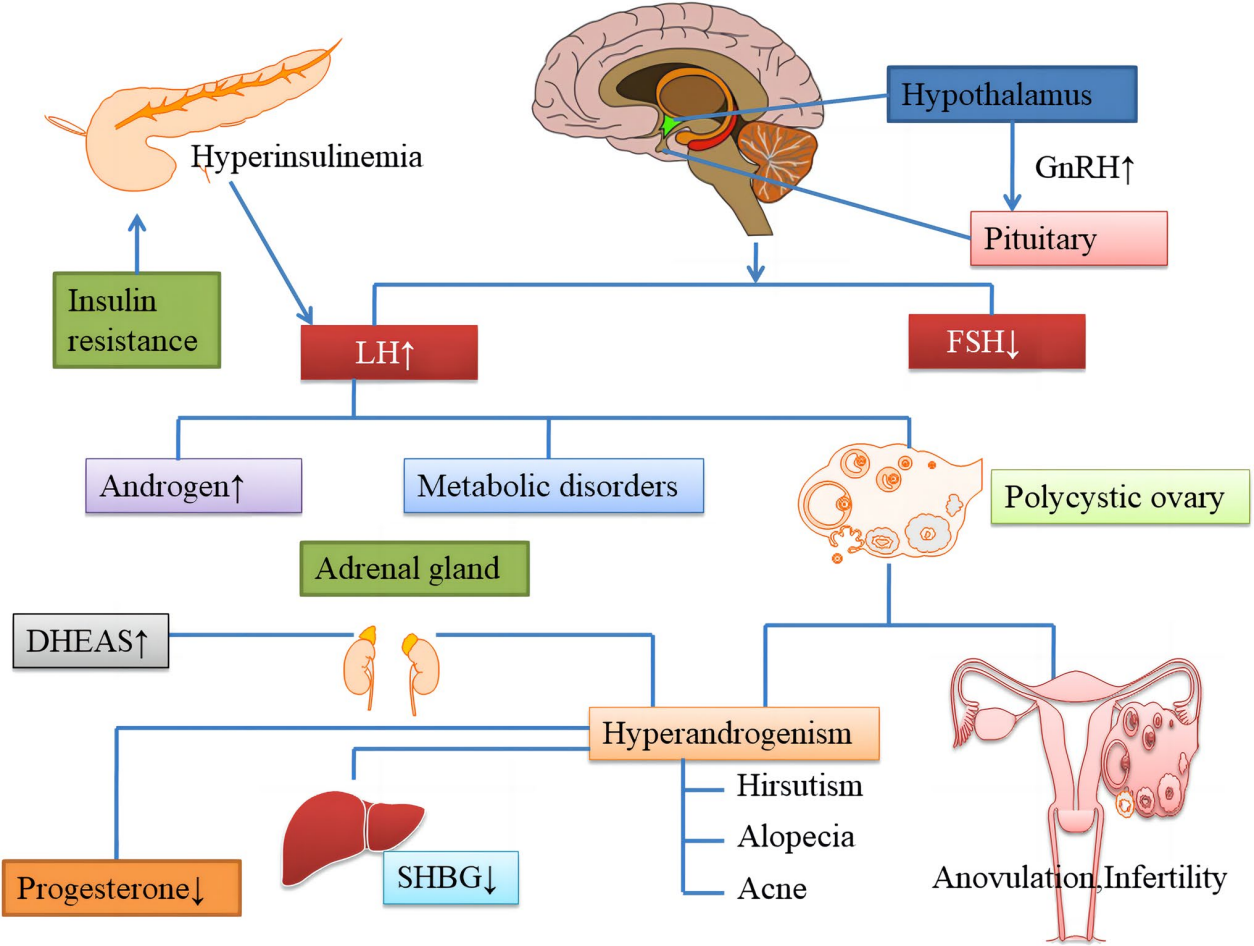
The pathophysiology of PCOS. Several theories have been proposed to explain the etiology of PCOS. Abnormal GnRH pulsation leads to an increase in the pulse frequency and amplitude of LH release and a relatively low release of FSH. This causes excessive androgen production, metabolic disorders, and other related performances. IR with hyperinsulinemia further increases ovarian androgen production directly and indirectly by inhibiting the production of SHBG by the liver. PCOS, polycystic ovary syndrome; GnRH, gonadotropin releasing hormone; LH, luteinizing hormone; FSH, follicle stimulating hormone

Globally, PCOS is the most common endocrine disease and one which causes female infertility [23]. Despite decades of research, the etiology and pathophysiological mechanisms of PCOS are as yet poorly understood [24–26]. Abnormal ovarian steroid production [27, 28], IR and hyperinsulinemia [29], and abnormalities in neuroendocrine control [30] are considered to be the main causes of PCOS. In most patients with PCOS, the pulse frequency of LH release increases and that of FSH release decreases, suggesting that GnRH pulse frequency is faster [31]. Conversely, high LH levels contribute to an increase in androgen secretion from the ovarian follicular membrane cells, whereas a decrease in FSH levels can disrupt follicular maturation and ovulation. PCOS results in increased secretion of GnRH/LH and a weaker response to exogenous estrogen and P4 [32], indicating that the negative feedback effect of steroid hormones on GnRH neurons is impaired [33]. Some neurotransmitter and neuropeptide receptors expressed in GnRH neurons directly regulate the release of GnRH, LH, and FSH [34]. Moreover, IR and compensatory hyperinsulinemia play a major role in the pathophysiology of PCOS. Excessive levels of insulin act synergistically with LH to stimulate the production of excessive levels of androgens. This inhibits the production of sex hormone-binding globulin (SHBG) by the liver [35] and increases the concentration of free testosterone. The pathophysiology of PCOS is depicted in Fig. 1.

### Effect of NPY on the reproductive system

**Fig. 2:**
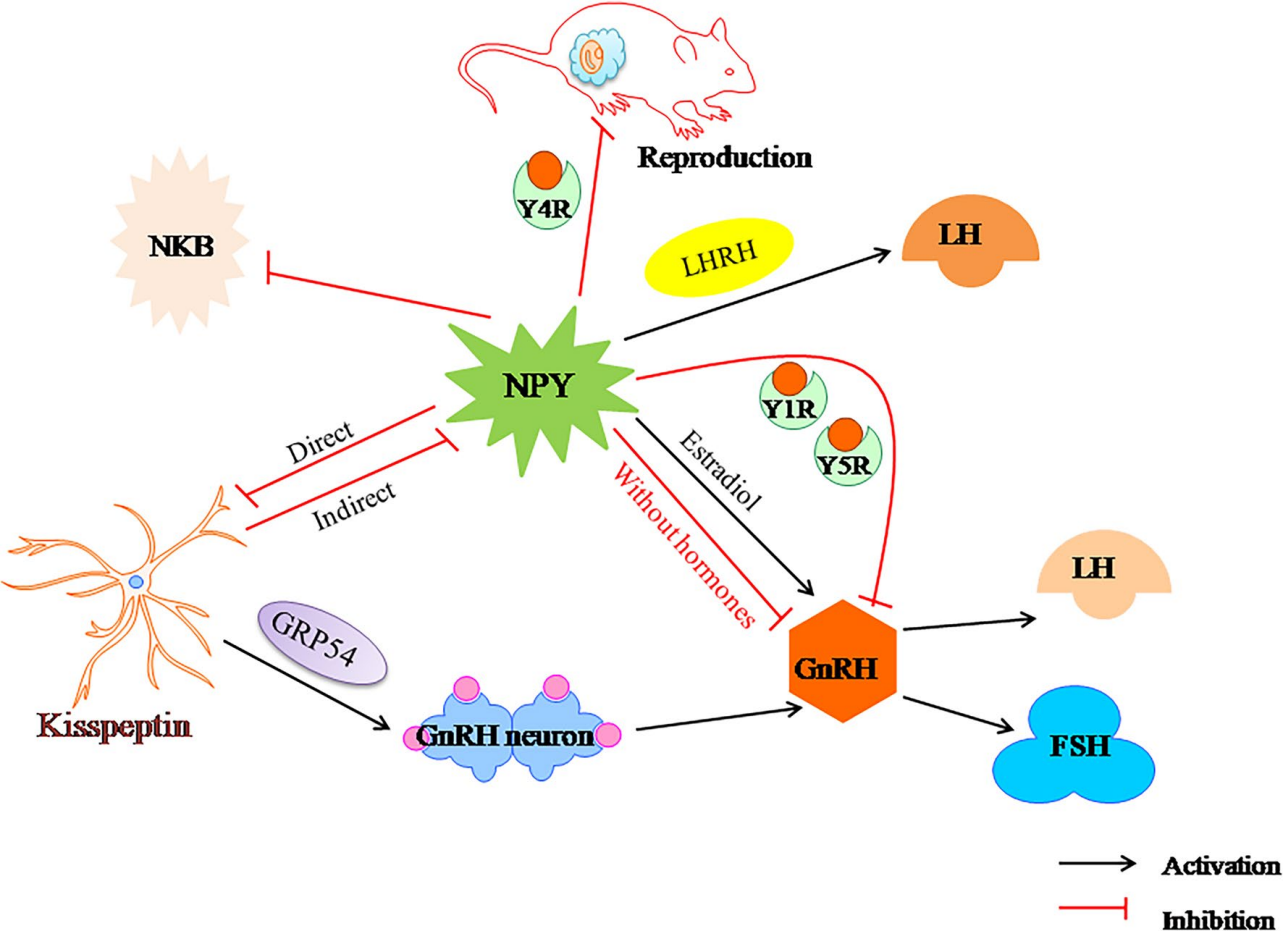
Relationship between NPY and fertility in polycystic ovary syndrome. NPY, neuropeptide Y; GnRH, gonadotropin-releasing hormone; LH, luteinizing hormone; LHRH, luteinizing hormone–releasing hormone; FSH, follicle stimulating hormone

Infertility is a common manifestation of PCOS, and approximately 90–95% of anovulatory infertility is caused by PCOS [5]. The reproductive system is controlled by the hypothalamic–pituitary–gonadal (HPG) axis [36], and depends on the proper functioning of the GnRH neuron network [23]. GnRH neurons secrete GnRH peptide into the pituitary portal system in timed pulses to promote the pulsating release of LH and FSH [37, 38]. Evidence suggests that NPY neurons have a negative effect on the HPO axis in female castrated animals [39, 40]. Results from prenatal androgen-induced sheep and mouse PCOS models suggest that an altered GABAergic input to GnRH neurons may play a role in the elevated GnRH/LH secretion [33, 41, 42]. Therefore, dysfunction of the GnRH neuronal network can lead to infertility [23]. NPY plays an important role in regulating the pulsatile release of GnRH [43], and regulates female reproductive function through the central nervous system [44]. The following section provides key evidence supporting the generally accepted effects of NPY on reproduction (Fig. 2).

### NPY stimulates LH release by regulating GnRH

NPY can act as a physiological stimulus to promote GnRH release before ovulation [13] affects the binding of GnRH and its receptors in the anterior pituitary of rats, and increases the response of anterior pituitary cells to GnRH release [43]. Notably, the fact that NPY enhances GnRH activity suggests that it interacts with GnRH to regulate LH secretion [45]. Evans et al. reported that NPY can regulate both the basal and GnRH-stimulated release of LH [45]. Moreover, NPY can also enhance GnRH-stimulated FSH secretion [46]. In ovary-intact ewes, the injection of NPY stimulates the release of GnRH in the follicular phase, but not in the luteal phase [47]. Additionally, Francis et al. found that the NPY-stimulated release of GnRH from the hypothalamus and of LH from the anterior pituitary requires normal ovarian function [48].

Interestingly, NPY can exert both stimulatory and inhibitory effects on GnRH neurons, and several studies have reported this to be steroid-dependent [49–51]. For example, NPY stimulates the release of GnRH in the presence of estradiol, but inhibits the release of GnRH in rats without sex hormones [52]. Coutinho et.al showed that arcuate nucleus neurons (ARN) NPY neurons inhibit GnRH/LH pulse frequency and decreased LH secretion in PCOS-like mice models [53].These inhibitory effects of NPY on reproductive function may lead to a decline in fertility in conditions of negative energy balance, such as food restriction or strenuous exercise, that are related to an increase in hypothalamic NPY expression. However, in a prospective study, women with PCOS had lower NPY levels than weight-matched healthy women [54]. Another study demonstrated that circulating NPY levels in obese and non-obese PCOS adolescents are significantly higher than those in healthy adolescents [55]. The reasons for the difference in results are unknown and may require more research.

### NPY receptors and GnRH

The NPY released from the hypothalamus regulates the activity of the GnRH neuronal system through the NPY receptor and the secretion of LH through the pituitary gland [56–60]. NPY stimulates LH-releasing hormone (LHRH) secretion by directly acting on LHRH neurons, which process is mediated by Y1-like receptors in vivo [29]. Moreover, NPY neurons participate in the LH surge by increasing the production of NPY and subsequently promoting the release of LHRH and/or enhancing its effects [29]. Sainsbury et al. found that when NPY expression in the hypothalamus increases under normal physiological conditions, the Y4 receptor causes a decline in reproductive capacity. Knocking out the Y4 receptor restored the reproductive capacity of ob/ob mice [21]. One study showed that NPY inhibits the excitability of GnRH neurons through the Y1 receptor and stimulates their excitability through the Y4 receptor [61]. The affinity of NPY for the Y4 receptor is 1000 × lower than that for Y1 receptor [62], indicating that endogenous NPY affects GnRH neurons via inhibitory events mediated by the Y1 receptor [61].

In addition, a study in lactating rats demonstrated that NPY exerts a direct inhibitory effect on GnRH neurons via postsynaptic Y5R [11]. Direct effects of NPY on proliferation and apoptosis of porcine ovarian cells have been reported [63]. Urata and colleagues found that the NPY5R in granulosa cells isolated from early antral follicles was significantly higher than that in late antral follicles [12]. Moreover, Pinilla et al. observed that Y2 receptors play a complex dual role in controlling gonadotropin secretion at various levels of the hypothalamic-pituitary unit in rats. PYY [13–36] (a selective Y2 receptor agonist) inhibits GnRH release. In contrast, the GnRH-stimulated response of gonadotropins is enhanced in the presence of PYY_13-36_ [59]. Table 1 provides a snapshot of the functions in which the NPY receptor is critical and the studies which support these findings. The above results may point to new strategies for using NPY receptors to improve neuroendocrine and ovarian function in PCOS patients.

**Table 1:** The major physiological roles of NPY receptors in humans, their agonists, and their antagonists

NPY receptors	Expression	Function	Agonist	Antagonist	References
Y1R	Periphery, hypothalamus, hippocampus, neocortex, thalamus	Food intake, energy homeostasis, body weight, angiogenesis, anti-anxiety, ethanol consumption, pain signaling, bone homeostasis, regulation blood pressure, sedation	[Leu31,Pro34]NPY, [Phe7,Pro34]pNPY, [D-Arg25]NPY, [D-His26]-NPY	1229U91,J-104870, J-115814,BMS-193885, SAR-135966,BVD-10, compound 3,BIBP3226, compound 4,SR120819A, BIBO3304	[14, 86, 124, 125]
Y2R	Brain, hippocampus, thalamus, hypothalamus	Food intake, energy homeostasis, colonic transit, pain signaling, cardiovascular regulation, anxiety, neuronal excitability, angiogenesis, ethanol consumption, bone formation	PYY(3–36),TM-30335, NPY13-36,obinepitide	BII20246,JNJ-31020028, JNJ-5207787(compound 7), T4-[NPY(33–36)]4	[14, 59, 86, 124, 125]
Y4R	Gastrointestinal tract, hippocampus, pancreas, hypothalamus, prostate, human epidermis	Food intake, energy homeostasis, regulates bone volume, body weight, affects fertility, muscle contraction	BVD-74D,1229U91, Sub[-T yr-Arg-Leu-Arg-T yr-NH2]2, TM-30339,obinepitide		[14, 21, 86, 124, 125]
Y5R	Hypothalamus	Food intake, energy homeostasis, anticonvulsant, anxiety, mood control	[D-Trp32]NPY, [D-Trp34]NPY, [cPP1-7, N P Y19-23, A l a31, Aib32, G l n34]hPP	CGP71683A,S-25585, GW438014A,MK-0557, FMS-586,L-152,804 velneperit (S-2367),	[14, 86, 124, 125]

### NPY inhibits kisspeptin

Kisspeptin is known to be a potent regulator of GnRH neuronal activity [1]. Several studies have confirmed that kisspeptin levels are increased in women with PCOS [64, 65] as well as in rodent models of PCOS [9]. However, kisspeptin levels were decreased in rats with dihydrotestosterone-induced PCOS, which conflicting results may be caused by different modeling methods [66]. Kisspeptin is a hypothalamic neuropeptide that drives fertility via the stimulation of GnRH neurons [67, 68] and induces the secretion of LH and FSH by directly activating GnRH neurons [67, 69, 70]. In addition, kisspeptin activates hypothalamic GnRH secretion through G-protein-coupled receptor GRP54 [71]. Abbara and coworkers demonstrated that kisspeptin receptor agonist (MVT-602) increases the firing duration of GnRH neurons and regulates LH levels, thus improving fertility outcomes in PCOS [72].

Sabine et al. reported that NPY had a direct inhibitory effect on a subpopulation of arcuate kisspeptin neurons in mice and suppressed neurokinin B-evoked firing. Fu and colleagues showed that kisspeptin inhibits NPY neurons through an indirect mechanism involving enhancement gamma-aminobutyric acid (GABA)-mediated inhibitory synaptic tone [73]. This indicates that NPY is negatively correlated with kisspeptin. However, the effect of NPY on kisspeptin neurons is still unclear [74], and, given the insufficient evidence in this regard, the subject requires further exploration.

Considering all the above, it is evident that NPY plays a significant role in the anovulatory infertility caused by PCOS, data which, on the other hand, potentially point to new strategies for PCOS infertility treatment. As present-day research is, however, primarily focused on animal models providing only limited clinical data, more studies are certainly needed.

## NPY affects metabolic disorders

The hypothalamus plays an essential role in the regulation of reproduction and energy balance [75]. Arcuate nucleus neurons coexpress NPY and agouti-related peptide (AgRP), which are key regulators of central energy homeostasis [76].

### NPY and obesity

Obesity is a growing global epidemic that creates both health and economic challenges [77, 78]. Weight gain and central obesity are the common features of PCOS, and usually occur before the start of the anovulatory cycle. Visceral obesity in PCOS patients is associated with elevated IR, which leads to an increase in reproductive disorders [79]. Obesity increases the risk for adverse metabolic and reproductive outcomes in patients with PCOS. Obesity also increases inflammatory adipokines, thereby promoting hyperinsulinemia, and amplifies functional ovarian hyperandrogenism by upregulating ovarian androgen production: this causes further weight gain, thereby forming a vicious feedback loop. Obesity increases IR and compensatory hyperinsulinemia, glucose intolerance, dyslipidemia, and the risk for pregnancy complications [80, 81]. The NPY system is hypothesized to play a key role in regulating energy balance and the pathophysiology of obesity [19].

Several studies have demonstrated that NPY regulates feeding behavior, body composition, and energy homeostasis and improves food efficiency, while it also induces food cravings and hormonal and metabolic changes and promotes fat gain [44]. In the arcuate nucleus, two types of neurons have opposite effects on food intake, namely, (1) neurons that coexpress NPY and AgRP and stimulate food intake (orexigenic), or those that can express insulin receptors [82, 83]; and (2) neurons that coexpress pro-opiomelanocortin (POMC) and cocaine and amphetamine-regulated transcript (CART), which restrain food intake (anorexigenic) [19, 44]. Consistent with its role as an orexigenic peptide, NPY is increased during fasting or calorie restriction, and is inhibited by feeding and the presence of leptin and insulin [84]. Sadeghian et al. found elevated NPY levels in obese women during a fasting mimicking diet [85]. In many cases of obesity, the elevated NPY-ergic tone may be due to central resistance to peripheral signals of energy excess such as leptin, which increases with long-term exposure to positive energy balance [86]. Similarly, Hansen et al. found that NPY synthesis is reduced in animals fed a high-fat diet [87]. Moreover, Baranowska et al. investigated the relationship between NPY levels and body weight and observed that plasma NPY levels increased significantly in both obese and non-obese patients with PCOS [29].

In addition, a number of studies have also demonstrated the importance of NPY receptors in mediating feeding responses. For example, specific ablation of Y2 receptors on NPY neurons led to a marked increase in obesity in female mice [88]. This was also the case in mice with conditional Y1 receptor knockout [89]. Moreover, the NPY Y5 receptor is also known to mediate NPY-induced feeding [90]. Fukasaka et al. demonstrated that NPY Y5 receptor antagonists significantly reduced weight gain and food intake [91]. Accumulating knowledge on the effect of NPY and its receptor on obesity may provide new insights into the treatment of obese women with PCOS.

### NPY and its effects on insulin resistance and hyperinsulinemia

IR and hyperinsulinemia are metabolic features characteristic of patients with PCOS and are considered an important component of the pathogenesis of this endocrine disease [92]. The prevalence of IR in PCOS patients is estimated to be 53–76% [24, 93]. IR is defined as decreased sensitivity of peripheral tissues to insulin. Therefore, higher insulin levels are needed to achieve its metabolic function, which leads to pancreatic β cells producing and releasing more insulin. This explains why IR is often associated with compensatory hyperinsulinemia [92, 94, 95]. Many studies have shown that the decrease of glucose transporter type 4 (GLUT-4) expression is one of the mechanisms underlying IR and PCOS [96–98]. Feng et al. found that insulin resistance reduced the expression of sex hormone-binding protein (SHBG) in human villous trophoblast cells, thereby inhibiting the expression of GLUT-4 and phosphatidylinositol 3-kinase (PI3K) p85α mRNA. This suggests that SHBG may be involved in PI3K/protein kinase B (Akt) pathway-mediated systemic insulin resistance [99]. In addition, IR and related hyperinsulinemia promote pituitary LH release, and increase testosterone production and SHBG synthesis, resulting in high levels of free testosterone (FT) [5, 100, 101]. On the other hand, IR stimulates GnRH gene transcription through the mitogen- activated protein kinase (MAPK) pathway in PCOS and increases LH secretion, thereby significantly increasing ovarian androgen synthesis [102].

Hyperinsulinemia and hyperandrogenism may promote the occurrence of acne. An observational study reported that the severity of acne in women with PCOS was associated with increased concentrations of FT and dehydroepiandrosterone sulfate [103]. High levels of insulin can lead to an increase in the concentration of insulin-like growth factor 1 (IGF-1). IGF-1 may stimulate the secretion of facial average sebum, increase the level of dehydroepiandrosterone sulfate, and induce the proliferation of sebocytes. In addition, hyperinsulinemia promotes the production of epidermal growth factor and transforming growth factor β, thereby increasing the level of non-esterified fatty acids in plasma, causing inflammation; it may thus lead to the colonization of epidermal bacteria in the follicles and the development of acne vulgaris [104]. Moreover, Kim et al. reported that insulin directly affects GnRH neurons, especially by stimulating GnRH gene expression to regulate reproductive function [105]. In summary, given that IR and hyperinsulinemia play a key role in PCOS and associated metabolic complications, targeting these disorders may prove to be beneficial in the treatment of this syndrome.

Sato et al. demonstrated that insulin played a role in reducing NPY gene expression in the hypothalamus. Insulin functions via neurotransmission, and the GABAergic system may also be involved in its effects on NPY neurons [106]. Singhal et al. reported that central resistin induced hepatic IR in mice through NPY [107]. Moreover, Hoek et al. reported that in rodents and humans fed a high-fat diet, increased levels of NPY in the hypothalamus may enhance glucose production and lead to sympathetic hyperactivity and hepatic IR [108]. Previous studies have shown that the activation of AgRP neurons induces IR partly through the acute suppression of sympathetic activation (SNA) in brown adipose tissue (BAT) [109]. However, the ability of AgRP neurons to induce IR depends on NPY expression. Consistent with this, intravenous cephalic injection of NPY rapidly and profoundly reduces BAT SNA [110, 111] and improves systemic insulin sensitivity [112]. In addition, prenatal exposure to androgens also reduces the colocalization of AgRP and insulin receptors. This may affect hepatic insulin sensitivity, as insulin in these neurons plays a prominent role in the regulation of hepatic glucose production [92]. Cernea et al. found that the decreased colocalization of IRβ in AgRP neurons may be a contributing factor to hyperinsulinemia and IR in adult ewes exposed to prenatal testosterone [113]. Interestingly, in patients with anorexia, leptin and insulin enter the brain, inhibit the activity of NPY/AgRP neurons, simultaneously stimulate the activity of POMC/CART neurons, and inhibit food intake [19]. The effects of leptin are discussed below.

### NPY and leptin

Leptin, a 167–amino acid polypeptide that is primarily synthesized and expressed in adipocytes [79, 114], is known to play an important role in energy homeostasis and reproduction. Leptin can induce anorexia and regulates energy requirements, fat reserves, and food intake. Insufficient energy intake (for instance, during fasting) leads to a decrease in leptin levels: this in turn stimulates intense hunger, causing an increase in food intake, which may subsequently lead to obesity [13]. Low leptin concentrations are an important signal of an energy deficit in the HPG axis, while high leptin concentrations in obese patients are usually associated with leptin resistance [115]. In patients with PCOS, plasma leptin levels are positively correlated with BMI [29]. NPY mRNA levels are increased in ob/ob mice but decrease after treatment with leptin. The knockout of NPY can attenuate obesity and other related symptoms in ob/ob mice, suggesting that NPY plays a role in the response to leptin deficiency [116]. In the brain, leptin regulates energy expenditure and other physiological functions through the leptin receptor (LepRb) [117, 118]. Zhang et al. showed that under high-fat diet conditions, NPY neurons’ lack of LepRb signaling leads to a significant increase in food intake accompanied by a decrease in energy expenditure, resulting in accelerated cellulite accumulation [119]. Leptin acts indirectly on kisspeptin neurons through POMC/CART and AgRP/NPY neurons to affect energy metabolism and GnRH release [75].

Leptin may also promote high androgen production by promoting steroid production and inhibiting NPY, leading to high levels of GnRH and LH [120]. The administration of leptin increases the levels of LH, FSH, and testosterone in fasting and ob/ob mice [114]. Barash et al. found that leptin specifically stimulates gonadal function in male and female ob/ob mice. Leptin treatment increased the weight of the ovaries and testes, and promoted follicular development in the ovary, which was consistent with the activation of ovarian function. Low levels of leptin stimulate the secretion of gonadotropins, whereas high levels of leptin have an inhibitory effect on the gonads. High levels of leptin have been shown to inhibit E_2_ synthesis and interfere with the formation of follicles, the production of steroid hormones, and the maturation of oocytes [121]. This suggests a potential for leptin therapy in patients with PCOS. However, the mechanism of leptin is still unclear and further research is needed.

## Conclusion and prospective directions

PCOS is a complex endocrine disease affecting reproduction, and metabolism [122]. It is a chronic lifelong disease that is a major health concern and poses an economic burden on patients and society [5]. Due to the complex etiology of the disease, the mechanism of its phenotypic development has not been fully elucidated. The classical theory suggests that the abnormal activation of hypothalamic GnRH neurons and ovarian androgen synthesis are involved in the core pathogenesis of PCOS [7]. In patients with PCOS, NPY not only regulates fertility by regulating GnRH/LH release and affecting the HPO axis, but also plays an important role in maintaining energy balance and regulating body weight and circulating glucose and lipid levels. In recent years, increasing numbers of studies have focused on the NPY receptors; however, the safety of this neuropeptide remains unclear, which precludes large-scale treatment of PCOS through its usage [123]. Therefore, further research should focus on exploring the safety of NPY application, determining what is the biological the mechanism of PCOS and identifying NPY receptors with high affinity and specificity as potential therapeutic agents.

## Abbreviations

AgRP: Agouti-related peptide
Akt: Protein kinase B
BAT: Brown adipose tissue
CART: Cocaine and amphetamine-regulated transcript
FSH: Follicle stimulating hormone
FT: Free testosterone
GABA: Gamma-aminobutyric acid
GLUT-4: Glucose transporter type 4
GnRH: Gonadotropin releasing hormone
HPG: Hypothalamic pituitary gonadal
HPO: Hypothalamic-pituitary-ovarian
IR: Insulin resistance
LH: Luteinizing hormone
LEpRb: Leptin receptor
LHRH: Luteinizing hormone releasing hormone
MAPK: Mitogen-activated protein kinase
NPY: Neuropeptide Y
PCOS: Polycystic ovary syndrome
P13K: Phosphatidylinositol 3–kinase
POMC: Pro-opiomelanocortin
PYY_13-36_: A selective Y2 receptor agonist
SHBG: Sex hormone-binding globulin
SNA: Sympathetic nerve activation
